# The Effects of *Crinum asiaticum* on the Apoptosis Induction and the Reversal of Multidrug Resistance in HL-60/MX2

**DOI:** 10.5487/TR.2008.24.1.029

**Published:** 2008-03-01

**Authors:** Jae-Hee Hyun, Jung-Il Kang, Sang-Cheol Kim, Elvira Kim, Ji-Hoon Kang, Jung-Mi Kwon, Doek-Bae Park, Young-Jae Lee, Eun-Sook Yoo, Hee-Kyoung Kang

**Affiliations:** 15 Department of Medicine, College of Medicine, Cheju National University, 66 Jejudaehakno, Jeju, 690-756 Korea; 25 Department of Veterinary Medicine, College of Applied Life Science, Cheju National University, Jeju, 690-756 Korea

**Keywords:** HL-60/MX2, *Crinum asiaticum*, Apoptosis, Chemosensitizer, MRP-1, BCRR

## Abstract

The present study investigated the anti-proliferative and chemosensitizing effects of *Crinum asiaticum* var. *japonicum* against multi-drug resistant (MDR) cancer cells. The 80% methanol extract, chloroform (CHCI_3_) fraction and butanol (BuOH) fraction of *C asiaticum* inhibited the growth of mitoxantrone (MX) resistant HL-60 (HL-60/MX2) cells. When HL-60/MX2 cells were treated with the CHCI_3_ and BuOH fractions, DNA ladder and sub-G1 hypodiploid cells were observed. Furthermore, the fractions reduced Bcl-2 mRNA levels, whereas Bax mRNA levels were increased. These results suggest that the inhibitory effect of *C. asiaticum* on the growth of the HL-60/MX2 cells might arise from the induction of apoptosis. Treatment of HL-60/MX2 cells with the fractions markedly decreased the mRNA levels of the multi-drug resistance protein-1 and breast cancer resistance protein. The CHCI3 fraction and hexane fraction increased MX accumulation in HL-60/MX2 cells. These results imply that the CHCI_3_ fraction of *C asiaticum* plays a pivotal role as a chemosensitizer. We suggest that components of *C asiaticum* might have a therapeutic potential for the treatment of MDR leukemia.

## INTRODUCTION

The development of multi-drug resistance (MDR) by tumor cells is a major obstacle to successful cancer chemotherapy (Riordan *et al*., 1979). MDR is the phenomenon by which exposure of tumor cells to a single cytotoxic agent results in cross-resistance to other, structurally unrelated, classes of cytotoxic compounds. MDR is multifactorial, and the strategies proposed to reverse MDR include targets from the apoptosis pathway, efflux transporters and so on. Increased expression of efflux proteins, which belong to the ATP-binding cassette (ABC) family of proteins, is a common feature of MDR (Allikmets *et al*., 1996; Goottesman *et al*., 1993), and overexpression of efflux proteins is associated with resistance to numerous anticancer agents (Larsen *et al*., 2000). The best known and well studied are P-glycoprotein (P-gp; MDR1) encoded by the *mdrl* gene (Endicott *et al*., 1989), multidrug resistance-associated protein-1 (MRP-1) encoded by the *mrp*1 gene (Cole *et al*., 1992), and breast cancer resistance protein (BCRP) encoded by the *abcg*2 gene (Doyle *et al*., 2003).

Another mechanism of MDR is related to resistance to apoptosis of multi-drug resistant cells induced by cytotoxic agents. The cellular molecules involved in this mechanism include anti-apoptotic proteins such as Bcl-2, which are overexpressed, and pro-apoptotic proteins such as Bax, which are down-regulated (Sellers *et al*., 1999).

Many studies have shown a link between tumor hypoxia and MDR. Under hypoxic conditions, the cytotoxicity of chemotherapeutic agents such as cisplatin, etoposide, bleomycin and mitomycin C, is reduced (Koch *et al*., 2003). Hypoxia-elicited chemotherapeutic resistance has been reported in a number of cell types, including fibroblasts, breast cancer cells, glioma cells and testicular germ cells (Koch *et al*., 2003; Kalra *et al*., 1993; Liang, 1996). Hypoxia-associated chemotherapeutic resistance is a broad phenomenon. Comerford *et*

*al.* have reported that MDR1 gene expression and subsequent functional P-gp expression are dramatically upregulated in a hypoxia inducible factor-1 (HIF-1)-dependent manner in response to hypoxia (Comerford *et al.*, 2002).

Studies of *Crinum asiaticum* var. *japonicum,* a member of the tribe Amarylidaceae, have described that it contains several phenanthridine alkaloids, triterpene alcohols and flavonoids (Sakurai *et al.*, 1976; Takagi *et al.*, 1997; Min *et al.*, 2001). Alkaloids isolated from the bulbs of Amaryllidaceae have shown various pharmacological effects, such as antiviral (Gabrielsen *et al.*, 1992), antimalarial (Likhitwitayawuid *et al.*, 1993), cytotoxic (Likhitwitayawuid *et al.*, 1993; Abdel-Halim *et al.*, 2004; Petti *et al.*, 1990; Xie *et al.*, 2004), anti-inflammatory (Samud *et al.*, 1999) and antineoplastic activities (Furusawa *et al.*, 1988), as well as effects on diseases of the nervous system (Cakici *et al.*, 1997). In particular, crinamine from *C. asiaticum* has been reported to induce apoptosis in hepatoma cancer cells (McNulty *et al.*, 2007) and inhibit HIF-1 activity (Kim *et al.*, 2006). In spite of chemotherapeutic resistance related to the activity of HIF-1, there are few reports on the anti-proliferative effect of *C. asiaticum* in chemotherapeutic agent resistant cells.

In this paper, we demonstrate that the 80% methanol (MeOH) extract and several solvent fractions of *C. asiaticum* decreased the survival rate of HL-60/MX2 cells by the induction of apoptosis, as well as having a chemosensitizing effect that could increase the intracellular accumulation of drug and decrease the expression of MRP-1 and BCRP.

## MATERIALS AND METHODS

***Preparation of extract from C. asiaticum.*** *C. asiaticum* var. *japonicum* was collected in March 2003 at Jeju Island, South Korea. *C. asiaticum* was washed in distilled water, dried at room temperature and ground into a fine powder. The dried plant powder (100 g) was extracted with 3 l 80% methanol (MeOH) at room temperature for 3 days and then the supernatant was concentrated under a vacuum. The resulting crude extract (20 g) was suspended in water (1 l) and successively partitioned with hexane (1 l × 3), chloroform ($CHCl_3$; 1 l × 3), ethyl acetate (EtOAc; 1 l × 3) and n-butanol (BuOH; 1 l × 3), to give hexane (497 mg), $CHCl_3$ (162 mg), EtOAc (200 mg), BuOH (1160 mg) and $H_2O$ (4800 mg) fractions.

***Cell culture.*** The HL-60/MX2 cell line is a mitoxantrone resistant derivative of the human acute promyeloid leukemia cell line HL-60. The clone designated HL-60/MX2 was approximately 35 fold less sensitive to MX than HL-60 parental cells. The cell line was obtained from the American Type Culture Collection (ATCC) and was grown in RPMI 1640 medium supplemented with 10% fetal bovine serum and penicillin/streptomycin (100 U/ml and 100 μg/ml, respectively) at 37°C in a humidified 5% $CO_2$ atmosphere.

***Cytotoxicity test.*** The effect of the 80% MeOH extract or the solvent fractions on the growth of HL-60/MX2 cell was determined by measuring the metabolic activity using a 3-[4,5-dimethylthiazol-2-yl]-2,5-diphenyltetrazolium bromide (MTT) assay (Carmichael *et al.*, 1987). The MTT assays were performed as follows: HL-60/MX2 ($2.5 \times 10^5$ cells/ml) were treated for 4 days with 20, 50, 100 μg/ml of the 80% MeOH extract or the solvent fractions. After incubation, 0.1 mg (50 μl of a 2 mg/ml solution) MTT (Sigma, Saint Louis, MO, USA) was added to each well and the cells were then incubated at 37°C for 4 h. The plates were centrifuged at 1000 rpm for 5 min at room temperature and the media was then carefully aspirated. 150 μl dimethylsulfoxide was then added to each well to dissolve the formazan crystals. The plates were read immediately at 540 nm on a microplate reader (Amersham Pharmacia Biotech., USA). All the experiments were performed three times and the mean absorbance values were calculated. The results are expressed as the percentage of inhibition that produced a reduction in the absorbance by the treatment of crude extract or solvent fractions compared to the untreated controls.

***Apoptosis assay.*** HL-60/MX2 cells ($2.5 \times 10^5$ cells/ml) were treated with 100 μg/ml of the 80% MeOH extract or the solvent fractions for 24 h. For the DNA fragmentation assay, the cells were collected by centrifugation and DNA was extracted using the Wizard genomic DNA purification kit (Promega, Madison, WI, USA). The DNA fragmentation pattern was analyzed by electrophoresis on a 1.5% agarose gel containing 0.1 μg/ml ethidium bromide for 40 min at 100 V (Oberhammer *et al.*, 1993). For the flow cytometric analysis to determine cell cycle phase distribution, the treated cells were washed twice with phosphate-buffered saline (PBS) and fixed in 70% ethanol for 30 min at 4°C. The cells were then rinsed with PBS and incubated in 50 μg/ml propidium iodide solution (PI; Sigma, Saint Louis, MO, USA) and 50 μg/ml RNase A in the dark for 30 min at 37°C. Flow cytometry analysis was performed using an EPICS-XL FACScan flow cytometer (Coulter, Miami, FL, USA). The DNA histograms obtained were analyzed to measure the proportion of sub-G1 hypodiploid cells (Sherwood *et al*., 1994).

***RNA isolation and RT-PCR analysis.*** Total RNA was extracted from cells using Tri-Reagent (MRC, Cincinnati, OH, USA) following the manufacturer's instructions. The RNA extraction was carried out in an RNase-free environment. RNA was quantified by reading the absorbance at 260 nm according to the methods described by Sambrook *et al* (Sambrook *et al*., 1989). The reverse transcription of 1 μg RNA was carried out with M-MuLV reverse transcriptase (Promega, WI, USA), oligo (dT) 18 primer, dNTP (0.5 μM) and 1 U RNase inhibitor. The polymerase chain reaction (PCR) was performed in reaction buffer [cDNA, 1.25 U Taq DNA polymerase (Promega, WI, USA), 3' and 5' primer 50 μM each and 200 mM dNTP in 200 mM Tris-HCl buffer, pH 8.4, containing 500 mM KCl and 1~4 mM $MgCl_2$]. The following primer pairs were used for the PCR reaction: 5'-TGCACCTGACGCCCTTCAC-3' as the sense primer and 5'-AGACAGCCAGGAGAAATCAAACAG-3' as the antisense primer for Bcl-2 (Monaghan *et al*., 1992); 5'-ACCAAGAAGCTGAGCGAGTGT-3' as the sense primer and 5'-ACAAAGATGGTCACGGTCTGCC-3' as the antisense primer for Bax (Miyashita *et al*., 1995); 5'-CTGGTTTGATGTGCACGATGTTGG-3' as the sense primer and 5'-TGCCAAGACCTCTTCAGCAGCTACT-3' as the antisense primer for MDR1 (Kioka *et al*., 1992); 5'-GACGGGAGCTGGGAATGC-3' as the sense primer and 5'-ACAACCTACTCCGGTGCC-3' as the antisense primer for MRP-1 (Cole *et al*., 1993); 5'-GTCAGCTGTGGAGCTGTTCGTAG-3' as the sense primer and 5'-CACAAGTGCTGTTGTCCGTTACA-3' as the antisense primer for BCRP (Zhou *et al*., 2002); 5'-TGTTACCAACTGGGACGACA-3' as the sense primer and 5'-AAGGAAGGCTGGAAAAAGAGC-3' as the antisense primer for $\beta_2$-MG (Xie *et al*., 2003). The PCR was performed with a DNA gene cycler (BIO-RAD, HC, USA), and the amplification was followed by 35 cycles of 94°C for 45 sec (denaturing), 60~65°C for 45 sec (annealing) and 72°C for 1 min (primer extension). The PCR products were electrophoresed on a 3.5% agarose gel.

***Chemosensitizing effect of C. asiaticum.*** To determine MX uptake, HL-60/MX2 cells (1.0 × $10^6$ cells/ml) were treated with 20, 50 and 100 μg/ml of the 80% MeOH extract or the solvent fractions of *C. asiaticum* in culture media for 30 min at 37°C. Then 5 μmol/l MX was added for a further 40 min incubation at 37°C, after which the cells were washed twice with ice-cold PBS and kept on ice until analysis. The measurement of cellular MX fluorescence were made using flow cytometry in which a focused argon laser beam (485 nm) excited the cells in a laminar sheath flow and their fluorescence emissions (547 nm) were collected to generate a histogram. Cellular fluorescence (x-axis) from intracellular accumulation of MX is plotted versus cell number (y-axis). In addition, HL-60/MX2 cells were treated with the solvent fractions of *C. asiaticum* in the presence of cyclospolin A or verapamil which are known to be chemosensitizers (List, 1996; Loor *et al*., 2002; Garrigues *et al*., 2002; Choi *et al*., 1998).

***Statistical analyses.*** The student's t-test and one-way ANOVA were used to determine the statistical significance of differences between values for a variety of experimental and control groups. Data are expressed as a mean ± standard deviation (SD) from three independent experiments performed in triplicate. P-values of 0.05 or less were considered statistically significant.

## RESULTS

***The effect of C. asiaticum on the growth of HL-60/MX2 cells.*** When HL-60/MX2 (2.5 × $10^5$ cells/ml) were treated with 20, 50, 100 μg/ml of the 80% MeOH extract or the solvent fractions for 4 days, we found that 100 μg/ml of the MeOH extract, the $CHCl_3$ fraction and

**Table 1.** Inhibitory effects of the 80% MeOH extract or several solvent fractions from *C. asiaticum* on the growth of HL-60/MX2 cells

| Concentration (μg/ml) | Inhibition (%) | | | | | |
|---|---|---|---|---|---|---|
| | 80% MeOH | hexane | $CHCl_3$ | EtOAc | BuOH | $H_2O$ |
| 20 | 9.4 ± 4.7* | 5.6 ± 4.6 | 15.7 ± 0.7* | 9.9 ± 1.4 | 25.4 ± 3.8* | 8.5 ± 3.9 |
| 50 | 23.0 ± 1.2* | 16.9 ± 3.7* | 38.3 ± 1.9** | 23.4 ± 2.2** | 52.5 ± 1.8** | 18.2 ± 3.5* |
| 100 | 39.7 ± 3.4** | 22.6 ± 2.0** | 53.4 ± 1.9* | 32.3 ± 3.1* | 73.2 ± 1.4** | 23.2 ± 3.2* |

HL-60/MX2 cells (2.5 × $10^5$ cells/ml) were treated with 20, 50 and 100 μg/ml of the 80% MeOH extract or the solvent fractions from *C. asiaticum* for 4 days and measured for viability by MTT assay. The results are expressed as the percentage of inhibition that produced a reduction in the absorbance by the treatment of crude extract or solvent fractions compared to the untreated controls. All experiments were performed in triplicate. Data are presented as a mean ± SD from three separate experiments. *$p < 0.05$, **$p < 0.01$ compared with the control.

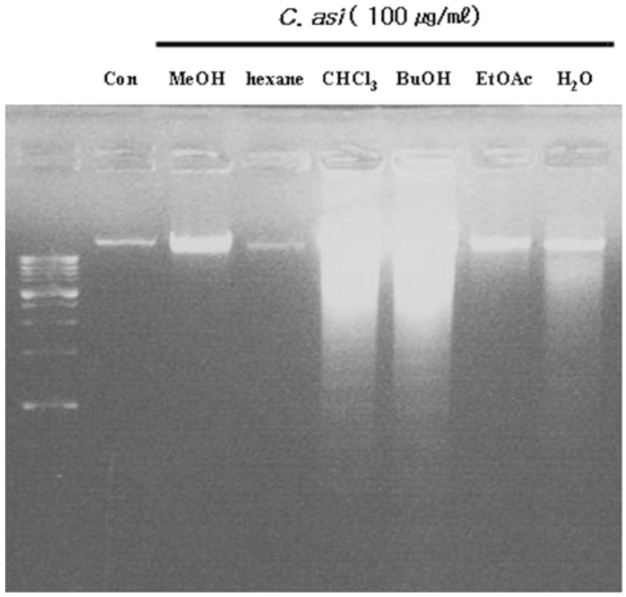

**Fig. 1.** DNA fragmentation by the 80% MeOH extract or several solvent fractions from *C. asiaticum* in HL-60/MX2 cells. HL-60/MX2 cells ($2.5 \times 10^5$ cells/ml) were treated with 100 μg/ml of the 80% MeOH extract or solvent fractions from *C. asiaticum* for 48 h. Analysis of DNA fragmentation was performed as described in materials and methods.

the BuOH fraction inhibited cell growth by 40%, 53% and 73% respectively (Table 1).

***The effect of C. asiaticum on induction of apoptosis in HL-60/MX2 cells.*** DNA fragmentation, a hallmark of apoptosis, was detected by the DNA agarose gel electrophoresis method (Fig. 1). DNA ladder bands were clearly detectable in HL-60/MX2 cells treated with the $CHCl_3$ fraction and the BuOH fraction. Furthermore, we quantified the cells in the sub-G1 population after treatment with the $CHCl_3$ fraction and the BuOH fraction by flow cytometry analysis. When treated with 100

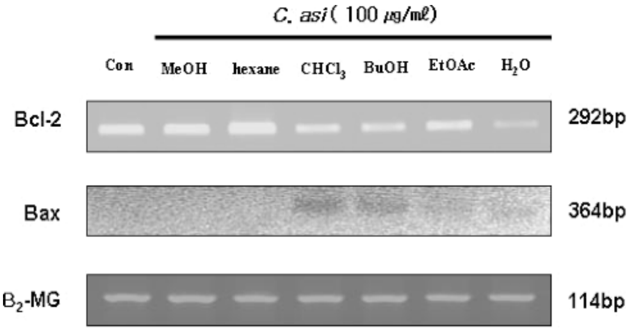

**Fig. 3.** RT-PCR analysis of Bcl-2 and Bax expression in HL-60/MX2 cells treated with the 80% MeOH extract or several solvent fractions from *C. asiaticum*. HL-60/MX2 cells ($2.5 \times 10^5$ cells/ml) were treated with 100 μg/ml of the 80% MeOH extract or the solvent fractions of *C. asiaticum* for 48 h. RT-PCR analyses of BCL-2 and Bax were performed after synthesizing the cDNA as described in the materials and methods.

μg/ml of the fractions for 24 h, the percentage of cells in the sub-G1 fraction increased (Fig. 2). To investigate the possible mechanism underlying the induction of apoptosis by *C. asiaticum*, we examined the expression of Bcl-2 and Bax in HL-60/MX2 cells after treatment with the MeOH extract or the solvent fractions of *C. asiaticum*. The expression of Bax increased markedly, while the expression of Bcl-2 decreased in the cells treated with 100 mg/ml of the $CHCl_3$ fraction or the BuOH fraction (Fig. 3).

***Chemosensitizing effect of C. asiaticum in HL-60/MX2 cells.*** To explore for the chemosensitizing effect of *C. asiaticum* in HL-60/MX2 cells, we examined whether *C. asiaticum* affected the intracellular accumulation of MX and mRNA expression levels of MDR-related genes. Flow cytometry revealed that the 80% MeOH extract, the hexane fraction, the $CHCl_3$ fraction and the BuOH

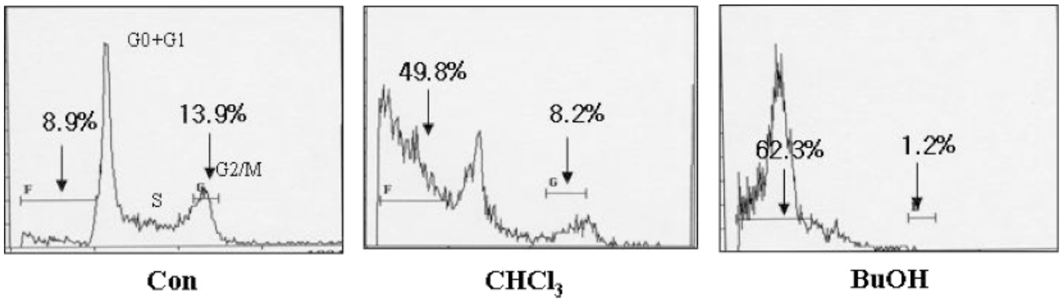

**Fig. 2.** The degree of apoptosis represented as the DNA content measured by flow cytometric analysis in HL-60/MX2 cells. HL-60/MX2 cells ($2.5 \times 10^5$ cells/ml) were treated with 100 μg/ml of the 80% MeOH extract, the $CHCl_3$ fraction and the BuOH fraction from *C. asiaticum* for 24 h. For the measurement of the sub-G1 hypodiploid cells, the flow cytometric analysis was performed as described in materials and methods.

**Table 2.** The effect of the 80% MeOH extract or several solvent fractions from *C. asiaticum* on MX accumulation in HL-60/MX2 cells

| Concentration (μg/ml) | Cell population for MX accumulation (%) | | | | | | |
|---|---|---|---|---|---|---|---|
| | Control | 80% MeOH | hexane | $CHCl_3$ | EtOAc | BuOH | $H_2O$ |
| 20 | 14.3 ± 1.6 | 19.3 ± 0.5** | 24.0 ± 0.3** | 45.8 ± 1.4** | 15.4 ± 0.3* | 15.7 ± 0.1** | 14.5 ± 0.2 |
| 50 | 14.3 ± 1.6 | 20.4 ± 0.3** | 24.4 ± 0.9** | 44.7 ± 0.2** | 14.8 ± 0.1* | 24.0 ± 0.2** | 13.8 ± 0.1 |
| 100 | 14.3 ± 1.6 | 33.2 ± 0.2** | 30.1 ± 0.2** | 46.1 ± 0.8** | 16.6 ± 0.3** | 42.7 ± 0.3** | 14.5 ± 0.1 |

HL-60/MX2 cells (1.0 × $10^6$ cells/ml) were treated with 100 μg/ml of the 80% MeOH extract or several solvent fractions from *C. asiaticum* and flow cytometry analysis of MX accumulation was performed as described in the materials and methods. The results are expressed as the percentage of cell population that produced cellular fluorescence by intracellular accumulation of MX. All experiments were performed in triplicate. Data are presented as a mean ± SD from three separate experiments. *$p < 0.05$, **$p < 0.01$ compared with the untreated control.

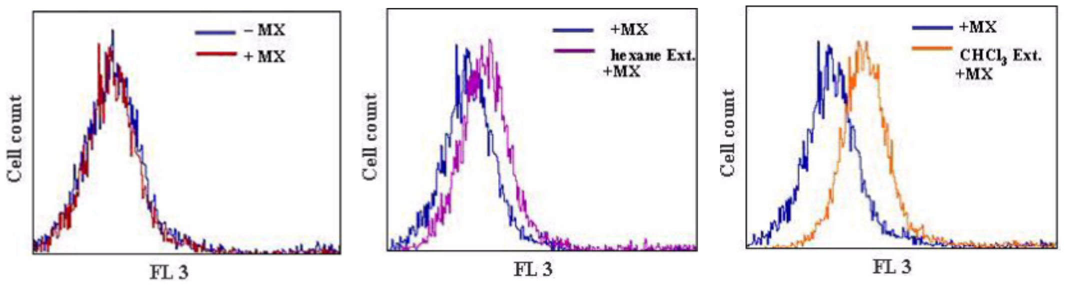

**Fig. 4.** The effect of the $CHCl_3$ fraction and the hexane fraction from *C. asiaticum* on the MX accumulation in HL-60/MX2 cells. HL-60/MX2 cells (1.0 × $10^6$ cells/ml) were treated with 100 μg/ml of the $CHCl_3$ fraction or the hexane fraction from *C. asiaticum* and flow cytometry analysis of MX accumulation was performed as described in the materials and methods.

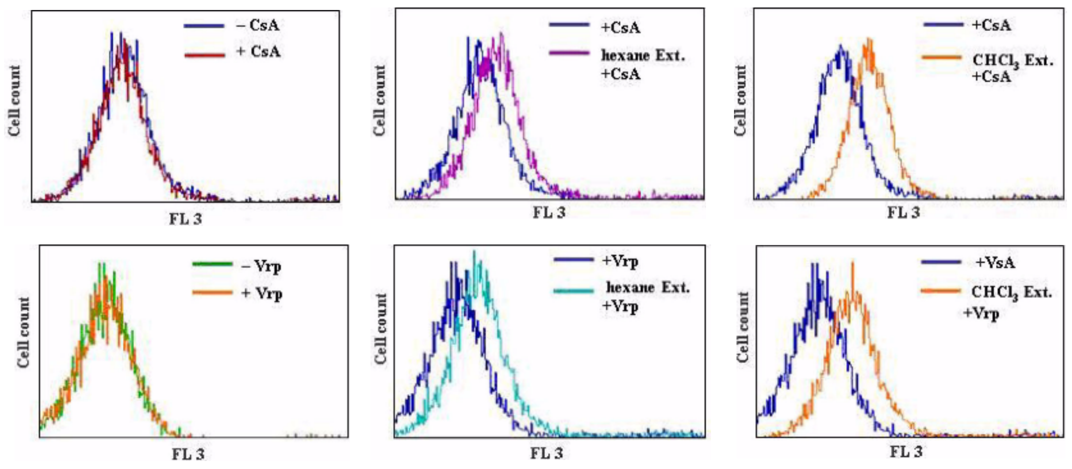

**Fig. 5.** The combined effect of cyclosporine A (CsA) or verapamil (Vrp) with the $CHCl_3$ fraction and the hexane fraction from *C. asiaticum* on MX accumulation in HL-60/MX2 cells. HL-60/MX2 cells (1.0 × $10^6$ cells/ml) were treated with 100 μg/ml of the $CHCl_3$ fraction or the hexane fraction from *C. asiaticum* in the presence of CsA or Vrp and flow cytometry analysis of MX accumulation was performed as described in the materials and methods.

fraction of *C. asiaticum* increased accumulation of MX in HL-60/MX2 cells (Table 2). Moreover, 100 μg/ml of the $CHCl_3$ fraction or the hexane fraction caused a right shift in the fluorescence peak associated with MX (Fig. 4). When HL-60/MX2 cells were treated with the $CHCl_3$ fraction or the hexane fraction of *C. asiaticum* in the presence of cyclospoline A or verapamil, known to be chemosensitizing compounds, no additive or synergistic effects on MX accumulation were observed (Fig. 5). We also investigated the expression of MDR1, MRP-1 and BCRP in HL-60/MX2 cells. The 295 bp mRNA of MDR1 was not detected in HL-60/MX2 cells. When HL-60/MX2 cells (2.5 × $10^5$ cells/ml) were treated with 100 μg/ml of the 80% MeOH extract or the solvent fractions

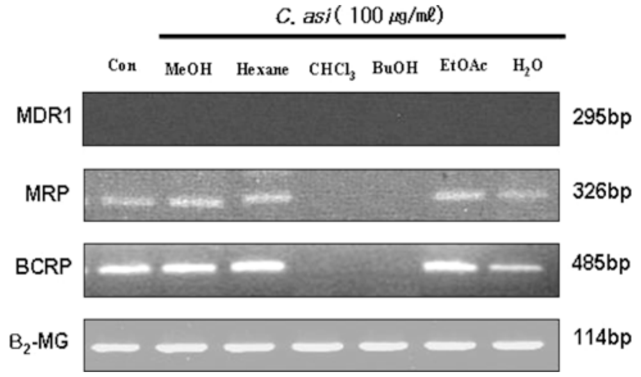

**Fig. 6.** RT-PCR analysis of MDR-1, MRP and BCRP expression in HL-60/MX2 cells treated with the 80% MeOH extract or several solvent fractions from *C. asiaticum*. HL-60/MX2 cells ($2.5 \times 10^5$ cells/ml) were treated with 100 μg/ml of the 80% MeOH extract or the solvent fractions from *C. asiaticum* for 48 h. RT-PCR analysis of MDR-1, MRP and BCRP were performed after synthesizing the cDNA as described in the materials and methods.

of *C. asiaticum* for 48 h, the $CHCl_3$ fraction and the BuOH fraction decreased the mRNA expression levels of MRP-1 and BCRP (Fig. 6).

## DISCUSSION

In present study, we examined whether *C. asiaticum* could overcome anti-cancer drug resistance in HL-60/MX2 cells. To the best of our knowledge, this study is first to show that an extract of *C. asiaticum* inhibited the proliferation of HL-60/MX2 cells by induction of apoptosis, increased MX accumulation and down-regulated MRP-1 and BCRP expression.

We observed that the 80% MeOH extract, the $CHCl_3$ fraction and the BuOH fraction from *C. asiaticum* decreased the survival rate of HL-60/MX2 cells. The extract also inhibited the proliferation of HL-60, but not HEL-299, which is a normal cell line (data not shown). The inhibitory effect of its extract on the growth of HL-60 cells might result from induction of apoptosis (*in submission*).

Strategies such as apoptosis induction, demethylation of genes affecting drug sensitivity, modulation of glutathione levels and increase of intracellular drug accumulation have been used to overcome MDR (Fojo *et al.*, 2003). Among these strategies, targeting Bcl-2, Bcl-$X_L$ and other proteins involved in apoptosis have appeared to be promising approaches to enhance chemotherapy efficacy (Fojo *et al.*, 2003; Yang *et al.*, 2003). Today, we recognize that apoptosis or programmed cell death is the outcome of a complex interplay of pro- and anti-apoptotic molecules. Pro-survival Bcl-2 and its helpers compete with Bax and other pro-apoptosis proteins to regulate the release of proteins and cytochrome c from mitochondria, which in turn activate 'initiator' caspases (Hanahan *et al.*, 2000). High levels of Bcl-2 expression have been found in several human tumors, and levels of Bcl-2 expression have been correlated with the aggressiveness of the malignancies (Pettersson *et al.*, 1992). Bcl-2 has been shown to block cytotoxic agents, whereas the inhibition of Bc1-2 function, by Bcl-2 antisense oligonucleotides (ASO) for example, precipitates apoptosis (Heere-Ress *et al.*, 2002). Since Bcl-2 functions by forming a heterodimer with its proapoptotic partner, Bax, the Bcl-2:Bax ratio is proportional to the relative sensitivity or resistance of the cells to various apoptotic stimuli (Oltvai *et al.*, 1993). Here, we showed that the $CHCl_3$ fraction and the BuOH fraction from *C. asiaticum* fraction reduced Bcl-2 mRNA levels, whereas Bax mRNA level increased. These results suggest that the inhibitory effects of *C. asiaticum* on the growth of HL-60/MX2 cells may arise from the induction of apoptosis via down-regulation of Bcl-2 levels. Crinamine was identified from BuOH fraction of *C. asiaticum* (Park, 2001) and reported to induce apoptosis in hepatoma cancer cells (McNulty *et al.*, 2007). In further study, it remains to be identified whether crinamine could induce apoptosis of HL-60/MX2.

Another approach that has been the subject of many studies is the use of chemosensitizers to modulate MDR chemo-resistant cells so that they become sensitive to chemotherapeutic agents. These chemosensitizers have a broad spectrum of chemical structures, which cause great difficulty in identifying the chemosensitizing properties of their structures. Several reviews have illustrated the required chemical structures of MRP-1 modulators (Boumendjel *et al.*, 2005). In this study, we examined the chemosensitizing properties of *C. asiaticum.* MX accumulation was increased in HL-60/MX2 cells when treated with the $CHCl_3$ fraction and the hexane fraction of *C. asiaticum*. The addition of cyclosporine A or verapamil, which are competitive inhibitors of MDR1 (List, 1996; Loor *et al.*, 2002; Garrigues *et al.*, 2002; Choi *et al.*, 1998), failed to show additive or synergistic effects on the accumulation of MX due to the $CHCl_3$ fraction and the hexane fraction of *C. asiaticum*. We found the expression of MRP-1 and BCRP, but not MDR1 in HL-60/MX2 cells as previously reported (Harker *et al.*, 1989). Active components from the $CHCl_3$ fraction and the hexane fraction of *C. asiaticum* might cause accumulation of MX via the inhibition of MRP-1 or BCRP. Furthermore, treatment of the HL-60/MX2 cells with the $CHCl_3$ fraction and the BuOH fraction resulted in marked decreases of MRP-1 and

BCRP mRNA levels. From these results, an active compound (or compounds) from the $CHCl_3$ fraction of *C. asiaticum* seems to play a pivotal role as a chemosensitizing mediator. The *C. asiaticum* has four alkaloids from BuOH fraction and two flavonoids from $CH_2Cl_2$ fraction (Park, 2001). The alkaloids were identified as (+)-crinamine, (5S,16S)-N-demethylgalanthamine, (5S,16R)-N-demethylgalanthamine and lycorine. The two flavonoids were identified as 4',7'-dihydroxy flavan and 4',7'-dihydroxy-4-methoxy chalcone. In particular, crinamine from *C. asiaticum* has been reported to induce apoptosis in hepatoma cancer cells (McNulty *et al.*, 2007) and inhibit HIF-1 activity (Kim *et al.*, 2006). Comerford *et al.* have shown that inhibition of HIF-1 expression resulted in significant inhibition of hypoxia-inducible MDR1 expression (Comerford *et al.*, 2002). The purified chemosensitizing principle from the $CHCl_3$ fraction of *C. asiaticum* remains to be identified and further studied for its mechanism of action in relation to HIF-1.

In conclusion, the $CHCl_3$ fraction of *C. asiaticum* might have cytotoxic effects on HL-60/MX2 cells by induction of apoptosis and chemosensitizing effects via the increase of MX accumulation and the down-regulation of MRP-1 and BCRP expression. The components of *C. asiaticum* should be studied in more detail in order to explain the molecular mechanism involved in apoptosis and modulation of efflux transporters connected with HIF-1.

## ACKNOWLEDGEMENT

This work was supported by a grant from the Cheju National University Hospital Research Fund (2006).
